# Supplementary material for: Global burden of Alzheimer’s disease and other dementias attributable to smoking in 204 countries and territories, 1990–2021
Source: PLoS One. 2025 Oct 15;20(10):e0334619. doi: 10.1371/journal.pone.0334619 (PMC12527123; doi:10.1371/journal.pone.0334619)
Supplement: Attachment — Submitted filename: Response letter to Reviewers.docx [file pone.0334619.s015.docx]

**Response letter to Reviewers by Dr. Wu et al** titled *“Global burden of Alzheimer's disease and other dementias attributable to smoking in 204 countries and territories, 1990–2021”*

PONE-D-25-28584

**Dear. Editor and Reviewers,**

Thank you very much for your interest in our manuscript entitled *“Global burden of Alzheimer's disease and other dementias attributable to smoking in 204 countries and territories, 1990–2021”*. To address the comments and concerns the Reviewers have raised, we have included a **point-by-point Response to each comment**. Within the revised manuscript, changes to the text in Response to the comments are marked as *red font*. And we revised the manuscript style in accordance with the *PLOS ONE.*

We appreciate the suggestions and comments by the Reviewers. As a consequence of these valuable suggestions, we believe that our manuscript has been much strengthened.

Please feel free to contact me if you and reviewers have further suggestions. I would be happy to make a revision according to their comments.

Yours sincerely

Dr. Wu

E-mail: [dr5rehab@163.com](mailto:dr5rehab@163.com)

# **Additional Editor Comments:**

**The manuscript has improved significantly. However, please carefully review it again for grammatical errors and duplicated content (e.g., lines 309–311 are repeated in lines 312–314)**

**Response**:

Thank you for your thoughtful feedback. I have carefully reviewed the entire manuscript and made thorough revisions to address the grammatical errors throughout the text. Additionally, I have removed the duplicated content, specifically the repeated sentences **(lines 337–339 in Revised Manuscript with Track Changes)**, to improve the clarity and flow of the manuscript. Your comments have been immensely helpful in enhancing the quality of this work.

# **Reviewer 1：**

1) **The changes to the manuscript announced in the response letter cannot be found in the corrected manuscript using the line numbers indicated as containing these changes. Examining the authors' responses is therefore difficult, if not impossible.**

**Response:**

I sincerely apologize for the incorrect line numbers I provided in the previous response; I realize this oversight made your second review far more difficult than it should have been. Although the revised manuscript has already been evaluated, I take full responsibility for the mistake and will implement a rigorous cross-checking procedure for all future submissions to ensure that every change is referenced accurately.

Thank you very much for your patience and for helping us improve the work.

2) **Upon reexamining the data, the authors discovered a large number of errors (over sixty) in Table 1. The authors do not state what led them to reexamine the data. They also state, on the one hand, that "We have comprehensively and thoroughly rechecked the raw data used for analysis and confirmed that the underlying source data is accurate and shows no anomalies," and on the other hand, they had to correct "related misinterpretations in** **Lines 285-290 of the Results section that stemmed from these initial data errors." It is therefore unclear whether the errors had an impact on the interpretation of the results.**

**Response:**

We sincerely apologize for the confusion caused by the inconsistencies. After the previous round of review drew our attention to a handful of minor descriptive figures elsewhere in the manuscript, we voluntarily undertook a line-by-line re-check of every numeric entry to ensure no similar transcription errors had occurred in any other part of the paper. During this self-initiated audit—conducted independently by three co-authors—we discovered that some values in Table 1 had been transposed (e.g., DALYs for two regions were swapped) when the analyzed data were copied from R into the manuscript. These mistakes were purely transcriptional; the underlying source files extracted from the GBD database remain intact and unchanged.

We immediately corrected Table 1 and the corresponding sentences **(****lines 218–226 in this version of Revised Manuscript with Track Changes)** in the revised manuscript submitted last round. We take full responsibility for this mistake and are deeply sorry. Fortunately, all subsequent analyses were conducted directly on the raw source files, so all figures and remaining reported results are correct.

To remove any remaining doubt, we now provide the raw datasets in supplementary materials:

**Smoking_AD.csv:** The original data downloaded from the GBD database includes locations such as the world, five SDI regions, seven super-regions, and 21 regions. The years range from 1990 to 2021.

**Smoking_AD_country.csv:** The "location" field covers 204 countries and regions.

**Smoking_AD_21Regions.csv:** The ASDRs values for 21 regions in 1990 and 2021.

**Smoking_AD_SuperRegionandGlobal.csv:** The ASDRs values for the global level, 5 SDI regions, and 7 super regions in 1990 and 2021.

Thank you for your patience and for holding us to the highest standards; we have instituted a mandatory double-entry verification protocol for all numeric content in future submissions.

3) **The main contribution of this manuscript is the consideration of DALY. This term therefore deserves to be better defined than by a simple equality which is not very understandable. The determination of DALY is more complex. Examples of calculation on concrete cases showing how the 2 terms (YLD and YLL) are obtained would be more informative. The interest of these examples would be to show how (and to what extent) the consideration of DALYs modifies the data.**

**Response:**

Thank you very much for highlighting the need to clarify DALY. We have now added a concise, step-by-step example (50-year-old smoker who develops AD and dies at 65) that shows how YLL and YLD are obtained and how their sum changes the perspective compared with mortality alone. We stress that real-world figures are produced with the full GBD modelling machinery and hope this revision makes the concept accessible to readers. We appreciate your guidance in improving the manuscript.

Please refer to **Lines** **128-144 in Revised Manuscript with Track Changes.**
